# Carbapenem-resistant Gram-negative pathogens in a German university medical center: Prevalence, clinical implications and the role of novel β-lactam/β-lactamase inhibitor combinations

**DOI:** 10.1371/journal.pone.0195757

**Published:** 2018-04-12

**Authors:** Juri Katchanov, Lucia Asar, Eva-Maria Klupp, Anna Both, Camilla Rothe, Christina König, Holger Rohde, Stefan Kluge, Florian P. Maurer

**Affiliations:** 1 Department of Intensive Care Medicine, University Medical Center Hamburg-Eppendorf, Hamburg, Germany; 2 Institute of Medical Microbiology, Virology and Hygiene, University Medical Center Hamburg-Eppendorf, Hamburg, Germany; 3 Division of Infectious Diseases and Tropical Medicine, First Medical Department, University Medical Center Hamburg-Eppendorf, Hamburg, Germany; 4 Hospital Pharmacy, University Medical Center Hamburg-Eppendorf, Hamburg, Germany; Emory University School of Medicine, UNITED STATES

## Abstract

**Objectives:**

To determine the spectrum of infections with multidrug-resistant Gram-negative bacteria (MDR-GNB) and the clinical impact of the newly available betalactam/betalactamase inhibitor combinations ceftolozane/tazobactam and ceftazidime/avibactam in a German academic tertiary care center.

**Methods:**

Retrospective analysis.

**Results:**

Between September 1, 2015 and August 31, 2016, 119 individual patients (0.22% of all hospital admissions) were colonized or infected with carbapenem-resistant MDR-GNB. The species distribution was *Pseudomonas aeruginosa*, n = 66; *Enterobacteriaceae* spp., n = 44; and *Acinetobacter baumannii*, n = 18. In 9 patients, carbapenem-resistant isolates belonging to more than one species were detected. Infection was diagnosed in 50 patients (total: 42.0%; nosocomial pneumonia: n = 23, 19.3%; bloodstream infection: n = 11, 9.2%). Antimicrobial treatment with broad-spectrum antibiotics prior to detection of a carbapenem-resistant isolate was documented in 105 patients (88.2%, prior administration of carbapenems: 62.2%). Nosocomial transmission was documented in 29 patients (24.4%). In 26 patients (21.8%), at least one carbapenem-susceptible, third-generation cephalosporin non-susceptible isolate was documented prior to detection of a carbapenem-resistant isolate belonging to the same species (median 38 days, IQR 23–78). 12 patients (10.1%) had documented previous contact to the healthcare system in a country with high burden of carbapenemase-producing strains. Genes encoding carbapenemases were detected in 60/102 patient isolates (58.8%; VIM-2, n = 25; OXA-48, n = 21; OXA-23-like, n = 10). Susceptibility to colistin was 94.3%. Ceftolozane/tazobactam and ceftazidime/avibactam were administered to 3 and 5 patients, respectively (in-hospital mortality: 66% and 100%). Development of drug-resistance under therapy was observed for both antimicrobials.

**Conclusions:**

i) The major predisposing factors for acquisition of carbapenem-resistant MDR-GNB were selective pressure due to preceding antimicrobial therapy and nosocomial transmission. ii) Colistin remains the backbone of antimicrobial chemotherapy for infections caused by carbapenem-resistant MDR-GNB. iii) Novel β-lactam/β-lactamase inhibitor combinations are of limited usefulness in our setting because of the high prevalence of Ambler class B carbapenemases and the emergence of nonsusceptibility under therapy.

## Introduction

Infections caused by multidrug-resistant Gram-negative bacteria (MDR-GNB) represent an increasing global threat [1]. Strains showing resistance to four major classes of antimicrobials (acylureidopenicillins, third generation cephalosporins, carbapenems and fluoroquinolones) pose a particular challenge due to limited therapeutic options [2]. Therapeutic regimens for infections caused by carbapenem-resistant MDR-GNB are often based on colistin, aminoglycosides, tigecycline and/or fosfomycin and their overall benefit on patient outcome is limited [3]. Recently, two novel β-lactam/β-lactamase inhibitor combinations, ceftolozane/tazobactam and ceftazidime/avibactam, have been approved by FDA and EMA for treatment of complicated urinary tract and intra-abdominal infections. *In vitro* and clinical trial data indicate substantial benefits of these antimicrobials, particularly against *Pseudomonas aeruginosa* isolates with up-regulated efflux and derepressed AmpC, and against *P*. *aeruginosa* and *Enterobacteriaceae* strains carrying class A (including KPC), some class C and D β-lactamases, respectively [4,5]. However, currently few data exist regarding the post-approval efficacy of ceftolozane/tazobactam and ceftazidime/avibactam in Europe and first reports on *a priori* and acquired non-susceptibility, mainly from the United States, raise concerns about their limited usefulness in the target population [6,7].

The aim of this study was to characterize patients colonized or infected with carbapenem-resistant MDR-GNB in a German academic tertiary care center. We sought to determine the frequency of isolation, source of origin, mode of acquisition as well as clinical manifestations. In addition, the impact of ceftolozane/tazobactam and ceftazidime/avibactam on the outcomes of selected, critically ill patients with infections due to carbapenem-resistant MDR-GNB was studied.

## Methods

### Study population

We conducted a retrospective, observational cohort study at the University Medical Center Hamburg-Eppendorf, a tertiary level medical center with 1656 hospital beds and approximately 75,000 admissions per year. All patients in whom at least one carbapenem-resistant MDR-GN bacterial isolate (reflecting either infection or colonization) was detected by culture in any specimen from 1^st^ of September 2015 to 31^st^ of August 2016 were included in the study. The following information was retrieved from electronic medical records (Soarian, Cerner, Idstein, Germany): age, sex, primary diagnosis, pre-existing medical conditions, presence of malignancy, preceding hospital stays as well as admissions to long-term care facilities, current and preceding anti-infective treatments, admissions to the ICU, travel and contacts to healthcare facilities in countries with high prevalence of multidrug-resistant bacteria and in-hospital mortality.

During the study period, a risk-adjusted screening scheme for MDR-GNB by rectal, pharyngeal and inguinal swabs was implemented. The indicators for screening were previous contact to health care facilities abroad and previous contact to patients with known colonization by MDR-GNB. In addition, all patients transferred to medical and surgical ICUs were screened upon admission. In case of clustered detection of single bacterial clones, additional screening samples were taken from all patients on the affected ward on a weekly basis.

### Microbiological data

Multidrug resistance was defined according to the recommendation by the national commission for hospital hygiene and infection prevention at Robert-Koch-Institute [8]. In brief, clinical *Acinetobacter baumannii*, *Pseudomonas aeruginosa* or *Enterobacteriaceae* isolates were classified as MDR in case of resistance to at least 3 out of 4 antimicrobial classes: i) acylureidopenicillins, ii) third and/or fourth generation cephalosporins, iii) imipenem and/or meropenem, and iv) ciprofloxacin. Information on species identification, phenotypic antimicrobial susceptibility testing (AST), and presence of carbapenemases was retrieved from the laboratory information systems of the microbiology service (Swiss Lab, Roche, Berlin, Germany; HyBASE, EpiNet AG, Bochum, Germany). Phenotypic AST was performed on the Vitek 2 platform (Biomérieux, Marcy l’Etoile, France) and interpreted according to the EUCAST clinical breakpoint table (version 7.0). Selected MICs including those for ceftolozane/tazobactam and ceftazidime/avibactam were determined by gradient diffusion tests (Biomérieux and Liofilchem, Roseto degli Abruzzi, Italy). Presence of the carbapenemase-encoding genes *bla*_KPC_, *bla*_NDM_, *bla*_VIM_, *bla*_IMP_, and, *bla*_OXA-48_ was assessed using a multiplex-PCR [9]. Identification of *bla*_OXA-23_ was performed according to the protocol of Woodford et al. [10]. Clonal identity of bacterial isolates was assessed by pulsed-field gel electrophoresis (PFGE) as described previously [11,12].

### Microbiological specimens

To analyze whether detection of carbapenem-resistant MDR-GNB in microbiological specimens represented infection or colonization, all specimens were grouped according to clinical relevance: (1) blood cultures, (2) intraoperative swabs and biopsies, (3) specimens, in which isolation of carbapenem-resistant MDR-GNB could be interpreted as colonization or infection (respiratory samples, urine), (4) superficial and screening swabs (pharyngeal, inguinal, rectal), which typically reflect colonization and not infection. Patients, in which MDR-GNB were detected in more than one specimen were assigned to the category with the highest clinical relevance.

### Ethics

The Ethics Committee of the Hamburg Chamber of Physicians approved the study protocol and waived the need to obtain consent for the collection, analysis, and publication of the retrospectively obtained and anonymized data for this non-interventional study.

## Results

During the study period (1st of September 2015 until 31st of August 2016), 119 patients colonized or infected with carbapenem-resistant MDR-GNB were identified, corresponding to a prevalence of 0.22% of all hospital admissions (54 225 unique patients). The median patient age was 58 years (IQR 47–67), 40 patients (33.6%) were female.

### Spectrum of pathogens and molecular mechanisms of carbapenem resistance

Carbapenem-resistant isolates of *P*. *aeruginosa* were detected in 66 patients (55.5%), *Enterobacteriaceae* in 44 patients (37.0%) and *A*. *baumannii* in 18 patients (15.1%). *Klebsiella pneumoniae* was the most frequently isolated species of carbapenem-resistant MDR *Enterobacteriaceae* (n = 29), followed by *Escherichia coli* (n = 6), *Enterobacter cloacae* (n = 4), *Enterobacter aerogenes* (n = 3), *Hafnia alvei* (n = 1) and *Klebsiella oxytoca* (n = 1). In 9 patients, carbapenem-resistant MDR-GNB belonging to more than one species were detected.

Multiplex-PCR for genes encoding KPC, VIM, NDM, IMP and OXA-48 carbapenemases was performed on 92 MDR-GN bacterial isolates from 85 individual patients (in 7 patients 2 isolates belonging to different species were tested). 50/92 isolates (54.3%) were PCR-positive while 42 isolates (45.7%) were PCR-negative. None of the tested isolates was positive for more than one carbapenemase. The most common carbapenemase was VIM-2 (n = 25), followed by OXA-48 (n = 21, Table 1). In patients with previous contact to foreign health care systems, the most frequently detected carbapenemases were OXA-48 (n = 4) and NDM-1 (n = 3) in *Enterobacteriaceae*, and VIM-2 (n = 1) in *P*. *aeruginosa*. In addition, 10 *A*. *baumannii* isolates from individual patients were tested for the presence of OXA-23-like carbapenemases. All isolates were positive (Table 1).

**Table 1:** Detection of carbapenemase alleles in clinical MDR-GNB isolates.

species	Serine-β-lactamases (Ambler Class A)	Metallo-β-lactamases (Ambler Class B)			Oxacillinases (Ambler Class D)	
	*bla*_KPC_	*bla*_NDM_	*bla*_VIM_	*bla*_IMP_	bla_OXA-23-like_	*bla*_OXA-48_
***Klebsiella pneumoniae* (n = 28)**	0	1 (3.6%)	0	0	n.a.	19 (67.9%)
***Klebsiella oxytoca* (n = 1)**	0	0	1 (100%)	0	n.a.	0
***Escherichia coli* (n = 6)**	0	2 (33.3%)	1 (16.7%)	0	n.a.	1 (16.7%)
***Enterobacter cloacae* complex (n = 4)**	0	1 (25.0%)	2 (50.0%)	0	n.a.	1 (25.0%)
***Enterobacter aerogenes* (n = 1)**	0	0	1 (100%)	0	n.a.	0
***Hafnia alvei* (n = 1)**	0	0	0	0	n.a.	0
***Pseudomonas aeruginosa* (n = 51)**	0	0	20 (39.2%)	0	n.a.	0
**Total (n = 92)**	0	4 (4.3%)	25 (27.2%)	0	n.a.	21 (22.9%)
***Acinetobacter baumannii* (n = 10)**	n.a.	n.a.	n.a.	n.a.	10 (100%)	n.a.

Abbreviations: n, number of tested isolates; n.a., not applicable.

### Patient characteristics

Clinical characteristics and preceding antimicrobial therapy of the affected patients are summarized in Table 2. An ICU stay within 6 months prior to detection of carbapenem-resistant MDR-GNB was documented in 68 patients (57.1%). In 36 patients (30.3%), the first detection of a carbapenem-resistant isolate was documented during an ICU stay. Antimicrobial therapy within 6 months prior to detection of carbapenem-resistant MDR-GNB was recorded in 105 patients (88.2%); 74 patients (62.2%) received carbapenems. Forty-five patients (37.8%) suffered from malignant disease (hematologic malignancies, n = 25; solid tumors, n = 23; both, n = 3).

**Table 2:** Clinical characteristics, prior antibiotic usage, and underlying conditions in 119 patients colonized or infected with carbapenem-resistant MDR-GNB.

characteristic	n (%)
preceding ICU stay within 6 months prior to detection of MDR-GNB	68 (57.1%)
first isolation of carbapenem-resistant MDR-GNB during ICU stay^1^	36 (30.3%)
≥ 3 hospital admissions within 6 months prior to detection of MDR-GNB	40 (33.6%)
preceding stay in long term care facilities	14 (11.8%)
antimicrobial therapy within 6 months prior to detection of MDR-GNB	105 (88.2%)
ureido-penicillins^2^	36 (30.3%)
cephalosporins, 3rd generation^2^	43 (36.1%)
carbapenems^2^	74 (62.2%)
fluoroquinolones^2^	36 (30.3%)
malignant disease	45 (37.8%)
hematologic malignancy	25 (21.0%)
solid tumor	23 (19.3%)
immunosuppressive drugs	32 (26.9%)
diabetes mellitus	27 (22.7%)
chronic kidney disease	22 (18.5%)
stem cell transplantation	16 (13.4%)
organ transplantation	11 (9.2%)
liver transplantation	5 (4.2%)
kidney transplantation	4 (3.4%)
heart transplantation	2 (1.7%)
congestive heart disease	12 (10.1%)
chronic wounds	12 (10.1%)
cystic fibrosis	8 (6.7%)
long-term Foley urine catheter	8 (6.7%)
liver cirrhosis	7 (6.0%)
long-term tracheostoma	6 (5.0%)
chronic obstructive pulmonary disease	6 (5.0%)

^1^Sample taken ≥ 48 hours after admission to ICU.

^2^Combination therapies were administered to several patients resulting in a cumulative percentage of > 100%.

### Affected wards and services

**Fig 1:**
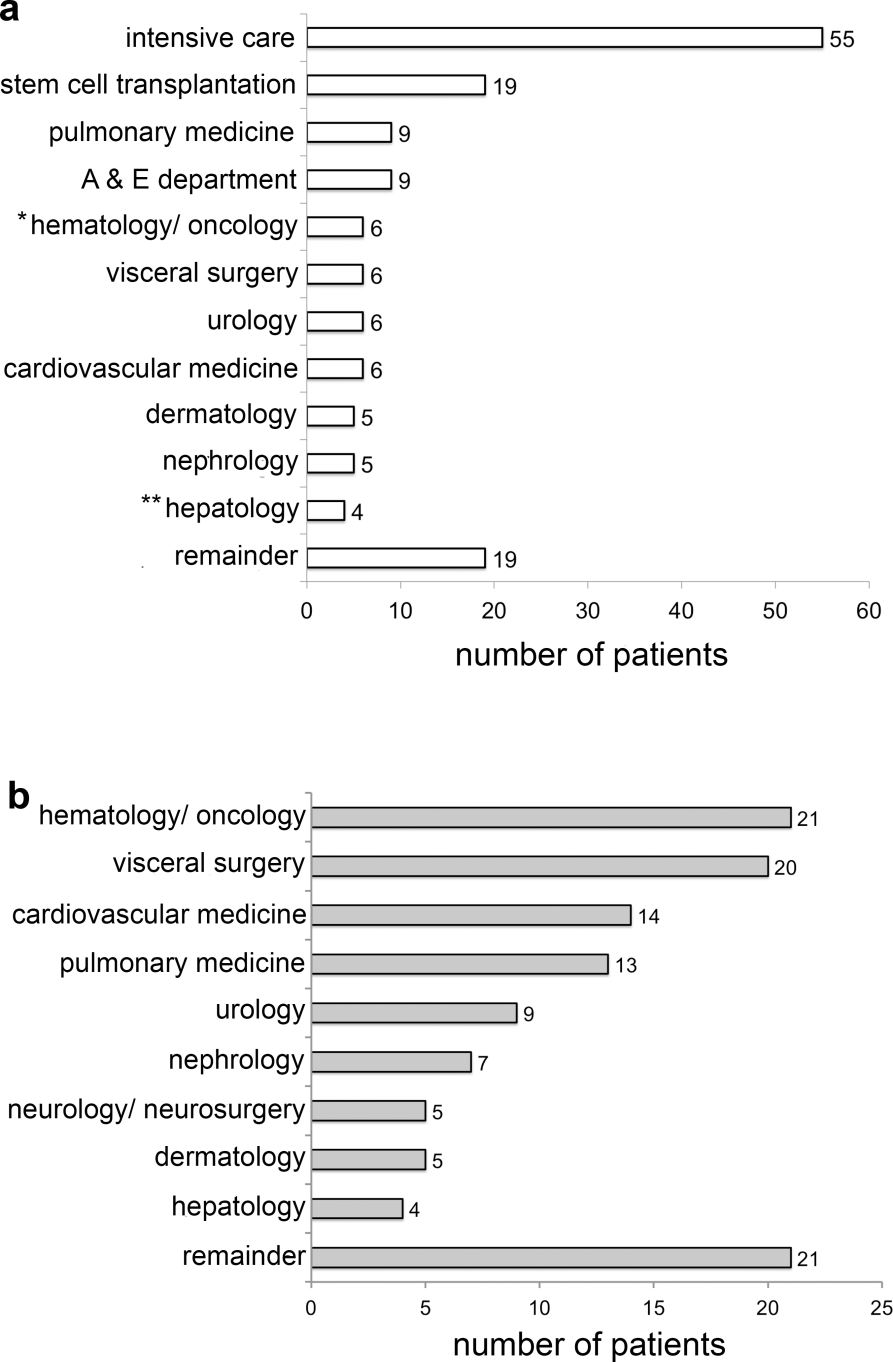
Distribution of MDR-GNB per hospital section. Panel A: Departments, in which carbapenem-resistant MDR-GNB were isolated. Each affected patient was counted once per department. Panel B: Services that primarily cared for these patients. Each patient was counted once per service. A & E, accident and emergency; *excluding stem cell transplantation ward; **including hepatobiliary surgery.

Fig 1A shows the distribution of hospitalized patients harboring carbapenem-resistant MDR-GNB per ward. The rate of detection was highest for ICUs (n = 55, 46.2%), followed by the ward for stem cell transplantation (n = 19, 16.0%). Fig 1B shows the services that cared for the affected patients on these wards. Twenty-one patients (17.6%) were associated with the hematology/oncology service, followed by 20 patients (16.8%), who were primarily treated by the department for visceral surgery. The three most frequent underlying conditions were hematologic malignancies (n = 19, 8 patients with acute myeloid leukemia), pulmonary disorders (n = 12, 7 patients with cystic fibrosis) and solid tumors (n = 11).

### Mode of acquisition

**Fig 2:**
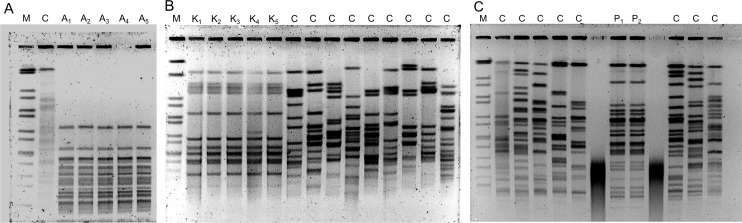
Examples of strain typing results using pulse-field gel electrophoresis. Panels A and B: Identical PFGE patterns generated from carbapenem-resistant MDR *Acinetobacter baumannii* (five individual patients A_1_-A_5_, panel A) and *Klebsiella pneumoniae* (five individual patients K_1_-K_5_, panel B) highly suggestive of nosocomial transmission. Panel C: Identical PFGE patterns of carbapenem-susceptible (P_1_) and carbapenem-resistant (P_2_) MDR *Pseudomonas aeruginosa* isolates from the same patient (blood culture and bronchioalveolar lavage, respectively) exemplifying acquisition of carbapenem-resistance under therapy. The time span between detection of the carbapenem-susceptible and carbapenem-resistant isolate was 21 days. Abbreviations: M, DNA Marker; C, unrelated clinical control isolate (same species, obtained outside the study period).

Identical PFGE patterns highly suggestive of nosocomial transmission were observed for 29 cases (24.4%; OXA-48-positive *K*. *pneumoniae*, n = 11 patients; OXA-23-positive *A*. *baumannii*, n = 10 patients; VIM-2-positive *P*. *aeruginosa*, n = 8 patients, Fig 2A and 2B). Twelve patients (10.1%) had documented contact to healthcare facilities in countries with high prevalence of multidrug-resistant organisms. In 26 patients (21.8%) a carbapenem-susceptible MDR-GN isolate was detected prior to detection of a carbapenem-resistant isolate belonging to the same species (*P*. *aeruginosa* n = 18 patients, *K*. *pneumoniae* n = 6 patients, and *E*. *coli* n = 2 patients). Clonal identity of corresponding isolates has been assessed in selected cases (Fig 2C). Emergence of carbapenem-resistance in these cases was on average observed after 38 days (IQR 23–78 days).

### Antimicrobial susceptibility testing for colistin, ceftolozane/tazobactam and ceftazidime/avibactam

**Fig 3:**
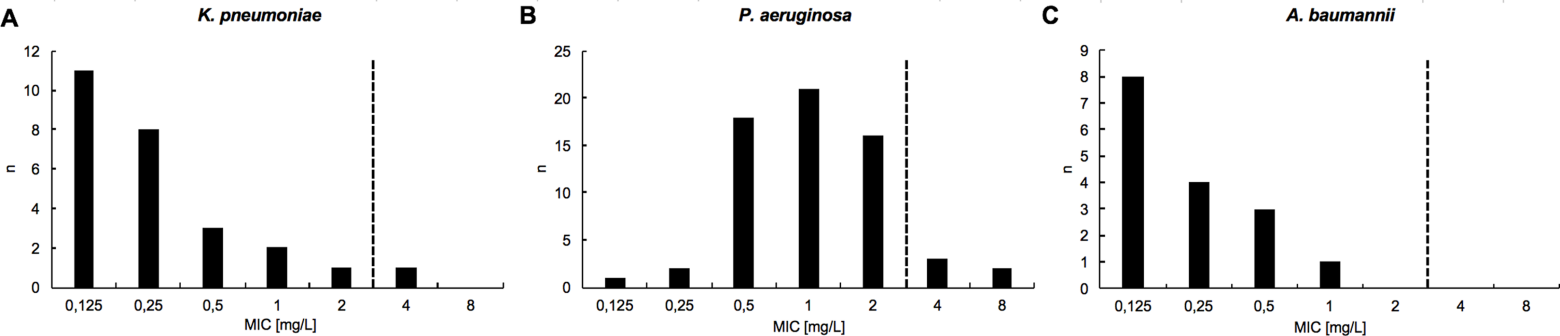
**Colistin minimal inhibitory concentrations (MICs) for a) *Klebsiella pneumoniae*, b) *Pseudomonas aeruginosa*, and c) *Acinetobacter baumannii* isolates.** Shown are non-duplicate isolates from individual patients. Dashed line: Clinical breakpoint according to EUCAST Breakpoint Table v.7.0 (MIC ≤ 2 mg/L, susceptible; MIC > 2 mg/L resistant).

AST for colistin was performed on 105 MDR-GN bacterial isolates. In total, 99/105 (94.3%) isolates were susceptible to colistin. Susceptibility was 96.2% for *K*. *pneumoniae* (25/26 isolates, Fig 3A), 92.1% for *P*. *aeruginosa* (58/63 isolates, Fig 3B), and 100% for *A*. *baumannii* (16/16 isolates, Fig 3C). MICs for ceftolozane/tazobactam were determined for carbapenem-resistant *P*. *aeruginosa* isolates from 20 individual patients. In 9 patients (45%) the MIC of the initial isolate was ≤ 4 μg/ml (susceptible). AST for ceftazidime/avibactam was performed for 15 isolates from 10 patients. In 5 patients with infections due to clonal Oxa-48 producing *K*. *pneumoniae*, ceftazidime/avibactam was administered based on initial MICs below ≤ 8 mg/L (susceptible).

### Colonization versus infection

Initiation of a specific antimicrobial regimen targeting carbapenem-resistant MDR-GNB, typically including administration of colistin, was used as a surrogate for diagnosis of probable infection as opposed to colonization. Table 3 shows the proportion of patients in whom infection by carbapenem-resistant MDR-GNB was diagnosed by the treating clinicians. Patients were grouped according to the sample source from which MDR-GNB isolates were obtained. In all but one patient with bacteremia due to carbapenem-resistant MDR-GNB, attending clinicians initiated targeted antimicrobial therapy (the remaining patient died before adequate therapy could be installed). For patients with positive culture results from non-sterile specimens only, e.g. respiratory samples and urine, diagnosis of infection was made in approximately 50% of cases. For patients with positive culture results from screening or superficial wound swabs only, targeted chemotherapy was initiated in up to 20% of cases.

**Table 3:** Distribution, initiation of treatment and in-hospital mortality of patients colonized or infected with carbapenem-resistant MDR-GNB.

Sample category	Likelyhood for infection	n of pts (% total)	treated for infection, n of pts (%)	in-hospital mortality (%)
**blood cultures**	very high	11 (9,2%)	10 (90,9%)	81,8%
**intraoperative swabs, tissue biopsies**	high	7 (5,9%)	5 (71,4%)	71,4%
**respiratory samples (TS, BAL, and sputum)**	equivocal	58 (48,7%)	28 (48,3%)	27,6%
**superficial swabs (wound swabs, ulcus swabs)**	low	9 (7,6%)	0 (0,0%)	0,0%
**routine screening swabs (nasal, rectal)**	very low	34 (28,6%)	7 (20,6%)	29,4%

Patients were stratified by the sample category from which carbapenem-resistant MDR-GNB were obtained. Patients in whom MDR-GNB were isolated from more than one specimen were counted once in the category with the highest likelihood for infection. Abbreviations: n of pts, number of patients; TS, tracheal secretion; BAL, bronchoalveolar lavage

### Clinical manifestations, treatment and outcome

For patients, in which detection of carbapenem-resistant MDR-GNB was associated with infection, the most common diagnosis was nosocomial pneumonia (n = 23). Bacteremia was diagnosed in 11 patients. In total, the attending physicians suspected infection and initiated antimicrobial therapy in 50 patients (42.7%). Total in-hospital mortality of the cohort was 33.6%. In-hospital mortality of patients with confirmed infection by MDR-GNB was 60.0%. Patient outcome stratified by specimen source is summarized in Table 3.

Colistin was administered to 45 patients (90% of all patients with clinically diagnosed infection). Ceftolozane/tazobactam and ceftazidime/avibactam were used mostly in combination with colistin or meropenem, in 3 and 5 critically ill patients with severe infections due to carbapenem-resistant MDR *P*. *aeruginosa* (no carbapenemase detected) and MDR *K*. *pneumoniae* harboring both Oxa-48 and a CTX-M-14 type extended-spectrum β-lactamase (ESBL), respectively (Table 4). In-hospital mortality of patients treated with ceftolozane/tazobactam and ceftazidime/avibactam was 66% and 100%, respectively.

**Table 4:** Clinical characteristics, microbiology data and outcome of patients treated with ceftolozane/tazobactam or ceftazidime/avibactam during the study period.

patient		1	2	3	4	5	6	7	8
**Age**		23	43	51	61	52	45	57	56
**Sex**		f	m	m	m	m	m	m	m
**underlying condition**		CF	lung transplant, PAH	liver transplant, SSC	esophago-tracheal fistula	AML	CHF, complicated duodeno-spleno-pancreatectomy (duodenal ulcer)	esophageal cancer, thoracoabdominal esophagectomy, gastric pull-up	chronic IBD
**infection due to** **MDR-GNB**		CAP/HAP	HAP	HAP, cholangitis	HAP	bacteremia	HAP	HAP	cIAI, HAP
**species**		*P*. *aeruginosa*	*P*. *aeruginosa*	*P*. *aeruginosa*	*K*. *pneumoniae*	*K*. *pneumoniae*	*K*. *pneumoniae*	*K*. *pneumoniae*	*K*. *pneumoniae*
**β-lactam** **resistance genes**		none detected	none detected	none detected	bla_OXA-48_, bla_CTX-M-14_	bla_OXA-48_, bla_CTX-M-14_	bla_OXA-48_, bla_CTX-M-14_	bla_OXA-48_, bla_CTX-M-14_	bla_OXA-48_, bla_CTX-M-14_
**Drug**		TOL/TZB	TOL/TZB	TOL/TZB	CAZ/AVI	CAZ/AVI	CAZ/AVI	CAZ/AVI	CAZ/AVI
**dose (g)**		1/0.5 q8h	1/0.5 q12h to 2/1 q8h	2/1 q8h	2/0.5 q8h	2/0.5 q8h	2/0.5 q8h	2/0.5 q8h	2/0.5 q8h
**total treatment duration (days)**		19	51 (2 courses)	12 (2 courses)	2	57	86 (4 courses)	8	61 (3 courses)
**MICs of last isolate before treatment (mg/L)**	**TOL/TZB**	1	2	0.38	16	>256	16	>256	>256
	**CAZ/AVI**	4	16	4	0,5	0.75	0.125	2	1
	**PIP**	>256	32	16	>256	>256	>256	>256	>256
	**MEM**	>32	>32	>32	>32	>32	>32	16	>32
	**CAZ**	32	16	16	>256	>256	4	>256	>256
	**CIP**	4	4	1	>32	>32	>32	>32	>32
	**COL**	0.125	1	0.75	16	0.25	0.25	2	16
**MICs of last available isolate (mg/L)**	**TOL/TZB**	>256	>256	8	n.a. n.a. n.a. n.a. n.a. n.a. n.a. n.a. n.a.	n.a. n.a. n.a. n.a. n.a. n.a. n.a. n.a. n.a.	12	n.a. n.a. n.a. n.a. n.a. n.a. n.a. n.a. n.a.	>256
	**CAZ/AVI**	>256	>256	16			0.75		32
	**PIP**	>256	>256	>256			>256		>256
	**MEM**	>32	>32	>32			>32		>32
	**CAZ**	>256	>256	32			4		>256
	**CIP**	>32	4	2			>32		>32
	**COL**	8	1	2			0.25		>16
**Outcome**	** **	discharged	died under therapy	died under therapy	died under therapy	died under therapy	died under therapy	died while hospitalized	died under therapy

Abbreviations: f, female; m, male; CF, cystic fibrosis; PAH, pulmonary arterial hypertension; SCC, secondary sclerosing cholangitis; AML, acute myeloid leukemia; CHF, congestive heart failure; IBD: inflammatory bowel disease; MDR-GNB, multidrug-resistant Gram-negative bacteria; CAP, community acquired pneumonia; HAP, hospital-acquired pneumonia; cIAI, complicated intra-abdominal infection; TOL/TZB, ceftolozane/tazobactam; CAZ/AVI, ceftazidime/avibactam; MIC, minimal inhibitory concentration; PIP, piperacillin; MEM, meropenem; CAZ, ceftazidime; CIP, ciprofloxacin; COL, colistin; n.a., not available

## Discussion

During the study period, carbapenem-resistant MDR-GNB were detected in 0.22% of all patients treated at our hospital. This frequency of isolation is in accordance with both data from another German academic medical center, where 0.3% of all hospitalized patients were colonized or infected with MDR-GNB, and with national data from the German nosocomial infection surveillance system (KISS, prevalence of 0.29% for carbapenem-resistant organisms in German ICUs in 2013–2014) [13]^,^[14]. In our study, *P*. *aeruginosa* was the most frequently isolated species showing an MDR phenotype with resistance to carbapenems, followed by *K*. *pneumoniae* and *A*. *baumannii*. This distribution is in line with other regional as well as national data from the KISS surveillance system [15,16].

More than half of the patients suffered from hematological malignancy, pulmonary disorders (cystic fibrosis), a visceral tumor or had a history of organ transplantation. Predisposition of these patient groups for colonization and infection with MDR-GNB is well described [17,18]. However, around 50% of patients with colonization or infection due to carbapenem-resistant MDR-GNB were treated in the departments of urology, neurology / neurosurgery, dermatology and others. Taking into account that the screening strategy was less stringent in these departments than on medical and surgical ICUs where every patient was screened upon admission, it is likely that the true prevalence of MDR-GNB outside these high-risk units is even higher. In conclusion, MDR-GNB pose a challenge to the hospital as a whole and are no longer restricted to selected departments. Nevertheless, ICUs still play a major role in acquisition and transmission of MDR-GNB [19–21]. In the present study, one third of the patient cohort acquired carbapenem-resistant MDR-GNB while in the ICU. The influx of colonized patients from foreign countries has been shown to be a relevant source of carbapenem-resistant bacteria in Germany [22–24]. However, in our study the number of directly imported cases was low as only approximately 10% of all patients had documented contact with healthcare facilities in countries with high prevalence of carbapenem-resistant MDR-GNB. In approximately 20% of patients a carbapenem-susceptible MDR-GN bacterial isolate had been documented prior to the detection of a carbapenem nonsusceptible strain belonging to the same species and identical PFGE patterns were highly suggestive of clonal identity. The majority of these isolates did not harbor any of the investigated carbapenemases. As most of the affected patients had been previously exposed to broad-spectrum antimicrobials, and in particular carbapenems, it is likely that other mechanisms such as upregulation or derepression of AmpC in combination with decreased uptake (porin loss) and/or increased efflux have caused the emergence of carbapenem resistance in these isolates [25].

The most frequently detected carbapemases in our study population were OXA-48, VIM-2 and OXA-23-like in *K*. *pneumoniae*, *P*. *aeruginosa* and *A*. *baumannii*, respectively. This distribution of carbapenemases is in line with data from the German National Reference Center for MDR-GNB [26]. Of note, the proportion of carbapenemase-expressing *P*. *aeruginosa* isolates in our study was approximately 30%, highlighting their growing importance in Germany [27].

Therapeutic options for infections with carbapenem-resistant MDR-GNB are limited [28,29]. Nearly all initial patient isolates in this study were susceptible to colistin and the compound is thus still the cornerstone of therapeutic regimens for carbapenem-resistant MDR-GNB [3,30,31]. However, a recent warning by EUCAST raises concerns regarding the accuracy of colistin MIC determination using widely adopted methods suitable for the routine diagnostic workflow such as gradient diffusion tests [32]. Further studies are required to investigate the impact of this observation on patient care.

The new β-lactam/β-lactamase inhibitor combination ceftolozane/tazobactam was resistant in 55% of all tested *P*. *aeruginosa* isolates. The high proportion of *P*. *aeruginosa* carrying VIM-2 carbapemases is a probable explanation for this phenomenon as ceftolozane/tazobactam shows no activity against Ambler class B (metallo-) carbapenemases [33]. In 2 out of 3 critically ill patients who received ceftolozane/tazobactam for treatment of pneumonia due to MDR *P*. *aeruginosa* expressing none of the investigated carbapenemases, development of high-level resistance (MIC >256 mg/L) was observed during therapy (Table 4). These findings corroborate previous data on clinical *P*. *aeruginosa* isolates indicating that resistance to ceftolozane/tazobactam can evolve through various combinations of AmpC overexpression, OprD porin loss and upregulation of the MexAB-OprM and MexXY-OprM operons[34]. One patient, a 23-year old woman suffering from cystic fibrosis, could nevertheless be discharged after 19 days of treatment. The second patient, a 43-year old man with a history of lung transplantation and hospital-acquired pneumonia, died after prolonged administration of ceftolozane/tazobactam for 51 days (in 2 courses). While it is likely that selection of a resistant bacterial subpopulation eventually led to the observed MIC increase, clinical deterioration upon cessation of ceftolozane/tazobactam after the first treatment course and the absence of alternative therapeutic options did necessitate re-administration of ceftolozane/tazobactam. The second novel β-lactam/β-lactamase inhibitor combination, ceftazidime/avibactam, was administered to 5 patients with severe hospital acquired pneumonia (and bacteremia in one case) during an outbreak of Oxa-48 and CTX-M-14 producing *K*. *pneumoniae* (Table 4). Similar to the above patients, prolonged administration of ceftazidime/avibactam for more than 10 days was required in three cases due to critical clinical condition. In one of these patients, a ceftazidime/avibactam MIC increase from 1 mg/L to 32 mg/L was observed under therapy. As has been shown in a separate study, a mutated CTX-M-14 isoform with augmented ceftazidime hydrolytic activity contributed to the emergence of ceftazidime/avibactam nonsuceptibility in this strain [35]. Finally, the large proportion of isolates expressing Ambler class B (metallo-) β-lactamases (Table 2) makes a combination of ceftazidime/avibactam and aztreonam particularly interesting for further investigations as aztreonam is stable against hydrolysis by class B enzymes and avibactam may protect aztreonam from hydrolysis by ESBLs, KPCs, and other cephalosporinases frequently found in the background of metallo-β-lactamase producing bacteria [36,37].

In our study, the decision to initiate treatment was influenced by the specimen category from which carbapenem-resistant MDR-GNB were recovered. It ranged from >90% in patients with positive blood cultures and around 50% in patients with positive cultures from non-sterile body sites (respiratory samples, urine) to 20% in patients with positive screening swabs only. These findings point to the difficulties of diagnosing infection due to MDR-GNB in patients with an extensive set of comorbidities. In our cohort, the in-hospital mortality of patients receiving specific treatment following detection of carbapenem-resistant MDR-GNB from screening swabs only was comparable to those with positive blood cultures (71.4% vs. 80%, respectively), indicating that the decision to diagnose infection and to initiate treatment without detection of MDR-GNB in clinically relevant samples is likely to be influenced by the severity of the patient’s overall clinical condition. This observation points to a potential bias when assessing both the efficacy of antiinfective therapy targeting carbapenem-resistant MDR-GNB and the mortality directly attributable to these pathogens.

This study has some limitations. It is a retrospective analysis and, hence, does not allow to draw firm conclusions on causal relationships between mortality and colonization/infection due to carbapenem-resistant MDR-GNB. While PFGE is a well-established method for outbreak investigations, current efforts are ongoing to allow more detailed investigations of clonal relationships between strains using whole genome sequencing data. To the best of our knowledge, the patients treated with the new β-lactam/β-lactamase inhibitor combinations were among the first in Germany. Accordingly, their overall number was low, and in the absence of prospective randomization, no final conclusions can be made with respect to the overall efficacy of ceftolozane/tazobactam and ceftazidime/avibactam in our setting.

## Conclusion

High numbers of patients colonized or infected with carbapenem-resistant MDR-GNB highlight the clinical relevance of these pathogens in German tertiary care centers. Notably, infections due to carbapenem-resistant MDR-GNB are no longer restricted to high-risk cohorts (hematological malignancy, pulmonary disorders, history of organ transplantation) but also increasingly affect patients in other departments. There is an urgent need for infectious diseases specialists coordinating their multidisciplinary management in close collaboration with hospital hygiene experts. Clinical microbiologists and pharmacists should be involved in managing these patients by overseeing rational design of antimicrobial regimens based on rapidly available phenotypic and genotypic drug susceptibility as well as therapeutic drug monitoring data. Fostering interdisciplinary management of infections due to MDR-GNB will facilitate timely initiation of effective therapies and prevention of unnecessary prescriptions of last resort antimicrobials.
